# Molecular characterization of misidentified *Plasmodium ovale* imported cases in Singapore

**DOI:** 10.1186/s12936-015-0985-8

**Published:** 2015-11-14

**Authors:** Jean-Marc Chavatte, Sarah Bee Hui Tan, Georges Snounou, Raymond Tzer Pin Valentine Lin

**Affiliations:** Malaria Reference Centre - National Public Health Laboratory, Ministry of Health, Singapore, 3 Biopolis Drive, Synapse #05-14/16, 138623 Singapore, Singapore; Sorbonne Universités, UPMC Université Paris 06, UPMC UMRS CR7, 75005 Paris, France; Centre d’Immunologie et de Maladies Infectieuses (CIMI) Paris, Institut National de la Santé et de la Recherche Médicale (Inserm) U1135, Centre National de la Recherche Scientifique (CNRS) ERL 8255, 75013 Paris, France; Department of Laboratory Medicine, National University Hospital, 5 Lower Kent Ridge Road, 119074 Singapore, Singapore; Department of Microbiology, Yong Loo Lin School of Medicine, National University of Singapore, 5 Science Drive 2, Block MD4, Level 3, 117545 Singapore, Singapore

**Keywords:** *Plasmodium ovale curtisi*, *Plasmodium ovale wallikeri*, Singapore, Imported cases, Misidentification, Morphology, Molecular characterization

## Abstract

**Background:**

*Plasmodium ovale*, considered the rarest of the malaria parasites of humans, consists of two morphologically identical but genetically distinct sympatric species, *Plasmodium ovale curtisi* and *Plasmodium ovale wallikeri*. These parasites resemble morphologically to *Plasmodium vivax* with which they also share a tertian periodicity and the ability to cause relapses, making them easily misidentified as *P. vivax*. *Plasmodium ovale* infections are rarely reported, but given the likelihood of misidentification, their prevalence might be underestimated.

**Methods:**

Morphological and molecular analysis of confirmed malaria cases admitted in Singapore in 2012–2014 detected nine imported *P. ovale* cases that had been misidentified as *P. vivax*. Since *P. ovale* had not been previously officially reported in Singapore, a retrospective analysis of available, frozen, archival blood samples was performed and returned two additional misidentified *P. ovale* cases in 2003 and 2006. These eleven *P. ovale* samples were characterized with respect to seven molecular markers (*ssrRNA*, *Potra*, *Porbp2*, *Pog3p*, *dhfr*-*ts*, *cytb*, *cox1*) used in recent studies to distinguish between the two sympatric species, and to a further three genes (*tufa*, *clpC* and *asl*).

**Results:**

The morphological features of *P. ovale* and the differential diagnosis with *P. vivax* were reviewed and illustrated by microphotographs. The genetic dimorphism between *P. ovale curtisi* and *P. ovale wallikeri* was assessed by ten molecular markers distributed across the three genomes of the parasite (Genbank KP050361-KP050470). The data obtained for seven of these markers were compared with those published and confirmed that both *P. ovale* species were present. This dimorphism was also confirmed for the first time on: (1) two genes from the apicoplast genome (*tufA* and *clpC* genes); and, (2) the *asl* gene that was used for phylogenetic analyses of other *Plasmodium* species, and that was found to harbour the highest number of dimorphic loci between the two *P. ovale* species.

**Conclusion:**

Misidentified *P. ovale* infections are reported for the first time among imported malaria cases in Singapore. Genetic dimorphism between *P. ovale curtisi* and *P. ovale wallikeri* was confirmed using markers from the parasites’ three genomes. The apparent increase of imported *P. ovale* since 2012 (with yearly detection of cases) is puzzling. Given decrease in the overall number of malaria cases recorded in Singapore since 2010 the ‘resurgence’ of this neglected species raises public health concerns.

**Electronic supplementary material:**

The online version of this article (doi:10.1186/s12936-015-0985-8) contains supplementary material, which is available to authorized users.

## Background

The formal description of *Plasmodium ovale* by Stephens in 1922 [1] was made more than 30 years after that of the other three species that naturally infect humans. However, its morphological resemblance to *Plasmodium vivax* delayed its acceptance as a *bona fide* species by many years [2, 3]. The clinical symptoms of primary *P. ovale* infections are typical of malarial infections (tertian high fever, chills, aches, and rigours) and the disease rarely progresses to severity although at least one fatal case was reported recently [4]. Peak parasitaemia is relatively low, and the infection often self-resolves, but with a tendency to re-appear even after effective treatment with schizontocidal drugs, as this species shares with *P. vivax* the ability to generate hypnozoites, dormant liver stages that activate weeks or months later and lead to a relapse episode [5]. Given the rarity of clinical severity, *P. ovale* has been used in malariotherapy for the treatment of neurosyphilis from 1930 to the 1960s [5–8], thereby providing data on the natural course of infection and the acquisition of immunity.

*Plasmodium ovale* occurs principally in West Africa and the Southwest Pacific where it can account for more than 10 % of all malaria infections. Outside these regions, its prevalence is generally quite low (<5 %, and often it is very rarely encountered), and its presence is yet to be confirmed in the Americas [9–16]. However, results from surveys based on sensitive molecular detection assays indicate that routine light microscopic examination of blood smears has underestimated its true prevalence [17–20]. In the course of such molecular surveys, a dimorphism in the 18S small sub-unit ribosomal RNA (ssrRNA) genes was discovered, and the otherwise morphologically similar *P. ovale* parasites were divided into ‘classical’ and ‘variant’ types [21, 22]. The dimorphism was later found to extend to other *P. ovale* genes, which led to the proposal that *P. ovale* is actually a species complex comprising two sympatric species: *Plasmodium ovale curtisi* (classic type) and *Plasmodium ovale wallikeri* (variant type) [23]. These two parasites have been observed in diverse endemic areas [24, 25], and the presence of a variant type was reported in a chimpanzee from Cameroon [26].

Singapore was officially declared free from malaria in November 1982 [27] after an assiduous anti-malarial programme initiated in 1911, which was only disrupted during World War II [28–30]. Nevertheless, Singapore remains highly vulnerable to the re-emergence of malaria due to the natural presence of competent vectors and the large number of foreigners arriving from malaria-endemic countries. Thus, 29 outbreaks of malaria have been reported in Singapore between 1983 and 2007 [31] and a focus of autochthonous transmissions of *P. vivax* confirmed in 2009 [32]. Furthermore, rare local zoonotic transmission of *Plasmodium knowlesi* has been recorded [33–35]. Surveillance and epidemiological monitoring of malaria cases are managed by the Ministry of Health (MOH) in close collaboration with the National Environmental Agency (NEA) which implements measures to control vector populations. The Malaria Reference Centre (MRC) relocated from the National University of Singapore (NUS) to the National Public Health Laboratory (NPHL) in 2010, along with the clinical malaria notification reports and their related blood smears collection (1994–2008), 226 frozen blood samples (collected from 2001 to 2008), and the submitted clinical malaria-positive samples from 2009. Since 2010, all notified malaria-positive clinical samples are sent directly to MRC-NPHL where a systematic species confirmation of all malaria-positive cases notified in Singapore is carried out both by classical morphology [36–38] and by a polymerase chain reaction (PCR) assay [39]. These data contribute to the malaria surveillance programme that aims to validate the routine morphological testing performed by the clinical laboratories, and to monitor any outbreaks or cases of re-introduction of malaria in Singapore.

Cases of *P. ovale* have never been officially reported in Singapore (Pr. Goh KT, pers. comm.), but examination of the reports from the country’s clinical laboratories from 1994 to 2014 revealed two cases: (1) a suspected *P. ovale* infection mixed with *Plasmodium falciparum* in 1998, although the morphological examination by the MRC was inconclusive at that time; and, (2) a suspected *P. ovale* infection in 2013 that was later confirmed to be *P. vivax* by MRC-NPHL. This report presents evidence of recently imported *P. ovale* infections in Singapore that had been misidentified as *P. vivax*, and includes a description of morphological and molecular characteristics of these parasites.

## Methods

### Samples from malaria-positive cases, Singapore (2001–2014)

From 2001 to 2014, a total of 2158 positive malaria cases, mainly imported, were notified to MOH by the clinical laboratories. For the period 2009–2014, a total of 830 positive malaria cases were directly received in MRC-NPHL and the infecting species was confirmed for all by morphology and by molecular methods. For the earlier period 2001–2008, archived frozen blood samples were available for 226 cases, and these were retrospectively tested as above. Thus, a total of 1,056 positive malaria cases recorded in Singapore were included in the present study. All the samples were collected for surveillance purpose for the MOH under the Infectious Diseases Act (Chapter 137), Part III-Control of Infectious Diseases within Singapore, section 7–Public Health Surveillance Programmes.

### Blood smears for microscopy

For routine surveillance, thick and thin blood films were prepared using standard procedures. Briefly, for thin smears one drop of blood was spread to a monolayer onto a glass slide using the edge of another glass slide, quickly air dried, fixed with absolute methanol and stained 20 min by 10 % Giemsa stain (Merck, Singapore) in pH = 7.2 phosphate buffer; for thick smears one drop of blood was smeared onto a glass slide, slowly and fully dried at room temperature or in an incubator, de-haemoglobinized in water and stained 8 min by 10 % Giemsa stain (Merck, Singapore) in pH = 7.2 phosphate buffer. Then all the smears were protected by a cover slip mounted with Eukitt ® (Sigma-Aldrich, Singapore) mounting medium and dried before reading. Blood smears were examined under ×1000 magnification with an Olympus CX31 microscope (Olympus, Singapore) and microphotographs taken with a Nikon Eclipse 80i microscope equipped with a Nikon DS Ri1 camera and the Nikon NIS Elements D Imaging Software (Nikon, Singapore).

### Blood DNA extraction

For routine surveillance, DNA was extracted using the EZ1® Advanced XL and the EZ1® DNA Blood 200 µL Kit (Qiagen®, Singapore) from 200 µL whole blood collected on EDTA following the manufacturers’ recommendations. The DNA template obtained was eluted in 200 µL stored at −30 °C until use.

### Identification of *Plasmodium* species by PCR

The protocol for routine surveillance includes two consecutive steps: first, a fast real-time PCR (rt-PCR) screen for the presence of malaria parasites by targeting a 186 bp fragment of the ssrRNA genes. The reactions are carried out using the StepOne™Plus Real-Time PCR System (Applied Biosystems®, Singapore), each in a final volume of 20 µL containing 10 µL of QuantiFast™ Probe master mix (Qiagen®, Singapore), 0.4 µM of each primer, 0.2 µM of each probe, 4 µL of nuclease free water, and 4 µL of DNA template, as previously described [40]. Samples found positive were then subjected to published nested-PCR (nt-PCR) assays that allow determination of the species, including *P. knowlesi* [39, 41]. All the reactions were run on Veriti® Thermal Cycler (Applied Biosystems®, Singapore) following the cycling conditions described in [39, 41] and PCR products were visualized after electrophoresis using the QIAxcel® Advanced instrument (Qiagen®, Singapore).

### Genetic characterization of *Plasmodium ovale*

In order to analyse genetic diversity, defined regions from ten different genes were amplified and then sequenced from all confirmed *P. ovale*-infected samples. All amplification reactions were run on Veriti® Thermal Cycler (Applied Biosystems®, Singapore) and PCR products were visualized after electrophoresis using the QIAxcel® Advanced instrument (Qiagen®, Singapore). The PCR products form all the assays were purified using the QIAquick® PCR Purification Kit (Qiagen®, Singapore) following manufacturers’ recommendations, eluted in 30 µL of nuclease free H_2_O and stored at −30 °C until use.

In order to achieve a fuller characterization of the *P. ovale* samples, genes belonging to the three genomes of the parasite were amplified and sequenced: (1) fragments of five nuclear genes that had been used previously to characterize the two *P. ovale* species: the 18S small sub-unit ribosomal RNA gene (*ssrRNA*), the reticulocyte binding protein 2 gene (*Porbp2*), the glyceraldehyde-3-phosphatase gene (*Pog3p*), the tryptophan rich antigen gene (*Potra*), and the dihydrofolate reductase thymidylate synthase gene (*dhfr*-*ts*), as well as a gene used for evolutionary studies of *Plasmodium* that had not been previously characterized for *P. ovale*, the adenylosuccinate lyase gene (*asl*); (2) fragments of two conserved genes from the mitochondrial genome commonly used for phylogenetic and evolution studies: the cytochrome c oxidase 1 gene (*cox1*) and the cytochrome b gene (*cytb*) were amplified and sequenced; and, (3) fragments of two conserved genes present on the apicoplast genome used for evolution studies that had not been previously characterized for *P. ovale*: the elongation factor Tu gene (*tufA*) and the caseinolytic protease C gene (*clpC*).

*ssrRNA* fragments were obtained through a nested PCR assay [39] with the primary amplification carried out with the rPLU1 + rPLU5 primer pair and the secondary reaction with the rPLU3 + rPLU2 primer pair, leading to the amplification of a fragment of about 862 bp [23, 25]. *Porbp2*, *Potra* and *Pog3p* fragments were obtained via a single PCR amplification as previously described [23] to yield fragments of 793 bp for *Porbp2*, from 293 bp to 365 bp for *Potra*, and 662 bp for *Pog3p*.

*dhfr*-*ts* fragments were obtained via a nested PCR amplification using a modified protocol based on previously published methods [23, 42]. The primary reaction is performed with one set of pan-*Plasmodium* primers (Pla-dhfrF–Pla-dhfrR) to yield a fragment of about 1925 bp, which was used as a template for secondary semi-nested amplifications performed with one of the primers used in the first amplification, paired with one *P. ovale*-specific oligonucleotide primers (Pla-dhfrF + Po5gsp2 and Po3gsp1 + Pla-dhfrR) to yield two overlapping fragments of about 870 bp and 1160 bp, respectively. Both products were sequenced to obtain the full *dhfr*-*ts* gene.

*asl* fragments were obtained with a newly developed single PCR amplification using one set of pan-*Plasmodium* oligonucleotide primers: asl-F (5′-CCMATCGAYGGGMGRTACAAA-3′) and asl-R (5′-TGTAAATTHCCYTCWGCATTTTC-3′) to obtain a fragment of about 915 bp. This amplification reaction is carried out in a total volume of 20 µL containing 1X High Fidelity PCR Buffer, 3 mM of MgSO_4_, 0.5 U of Platinum® *Taq* DNA Polymerase High Fidelity (Invitrogen™, Singapore), 0.2 mM of each dNTP (Promega, Singapore), 0.25 µM of each primer and 1.5 µL of the original DNA template. After an initial denaturation at 95 °C for 3 min, the cycling conditions were 94 °C for 30 s, 53 °C for 1 min and 68 °C for 1 min followed by a final extension at 68 °C for 10 min.

*cox1* fragments were obtained using a nested PCR protocol as previously described [26], to yield a fragment of about 1412 bp. *Cytb* fragments were obtained using a nested PCR protocol as previously described [43], to yield a fragment of about 1175 bp.

*tufa* fragments were obtained using a nested PCR protocol as previously described [44], to yield a fragment of about 814 bp. *clpC* fragments were obtained using a single PCR amplification as previously described [44], to yield a fragment of about 647 bp.

### Sequencing

The purified PCR products were sequenced in both direction using the BigDye® Terminator v3.1 cycle sequencing Kit (Applied Biosystems®, Singapore) using the appropriate oligonucleotide primers, following manufacturers’ recommendations. Then the BigDye® reaction’s products were purified using the BigDye® XTerminator™ Purification Kit (Applied Biosystems®, Singapore) following manufacturers’ recommendations before being sequenced on a 3500 × l Genetic Analyzer (Applied Biosystems®, Singapore).

### Sequences analysis

Alignment and cross-checking of the sequences were performed with CLC Main Workbench 5.7 software (CLC bio–Qiagen®, Singapore. Multiple alignments of the sequences with published sequences from GenBank were created to study the polymorphism between the two *P. ovale* species at key loci for each gene.

### Phylogenetic analyses

Molecular phylogenies were derived from a ≈800 bp fragment of the *asl* gene by maximum likelihood (ML) method with GTR + I +Γ model using PhyML software and by neighbour joining (NJ) method with J-C model using BioNJ algorithm. Nodal robustness of the tree was evaluated by non-parametric bootstrapping (1000 replicates).

## Results

### Detection of imported *Plasmodium ovale* cases in Singapore

Among the 316 cases notified to MOH by the clinical laboratories for the 2012–2014 period, nine cases reported as *P. vivax* have been re-identified as *P. ovale* by the MRC-NPHL (2012 n = 4, 2013 n = 3 and 2014 n = 2). As a consequence of these misidentifications, the MRC-NPHL conducted a retrospective study using the same combined morphological and molecular approaches on the 226 available malaria-positive, frozen blood samples collected between 2001 and 2008 in Singapore, and on samples from all the cases (n = 511) recorded during 2009–2011. This test returned a further two *P. ovale*-positive cases, one in 2003 and one in 2006, both misdiagnosed as *P. vivax*. Details of the reports and case histories for all known *P. ovale* cases in Singapore are presented in Additional file 1. To date, a single mixed infection case (not reconfirmed) and 11 pure infection *P. ovale* cases have been identified in Singapore.

### Morphological diagnosis

All the confirmed *P. ovale* cases in Singapore have been misidentified as *P. vivax* by the routine morphological examination performed at the hospital laboratories (Additional file 1). Thus, it seems important and useful to include a description of the erythrocytic stages based on [1, 7, 9, 45].

#### Description

The merozoite of *P. ovale* preferentially invades a reticulocyte, hence most of the young stages of this parasite are found in young red blood cells (RBCs).

#### Ring (Fig. 1a–c)

**Fig. 1:**
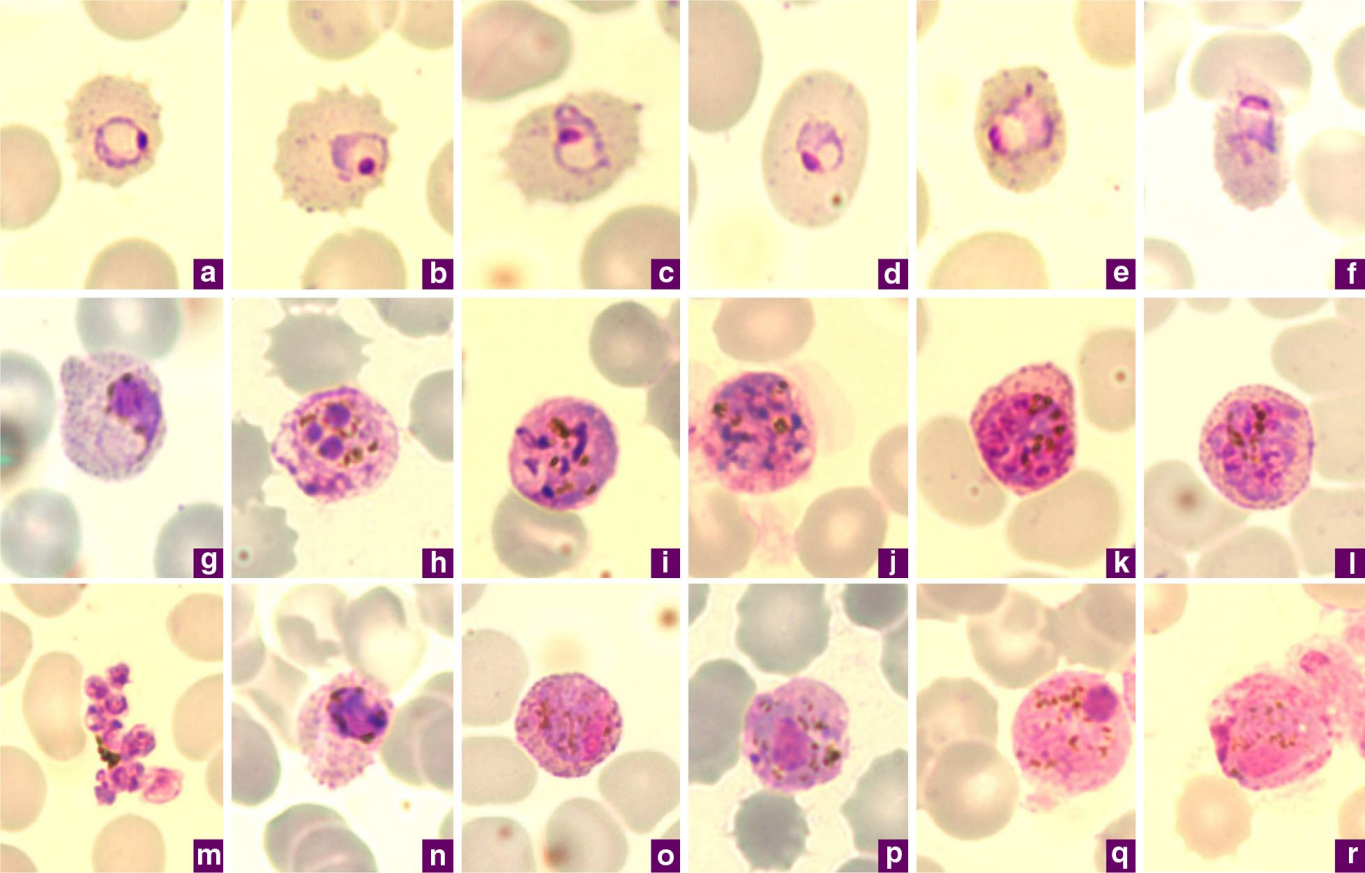
Microphotographs of *Plasmodium ovale* in Giemsa-stained thin blood films collected in Singapore. **a**–**c** ring stages, **d**, **e** young trophozoites, **f** trophozoite, **g** late trophozoite, **h** young schizont, **i**–**k** growing schizont, **l** late schizont, **m** ruptured schizont, **n** young gametocyte, **o**, **p** developing macrogametocytes, **q** macrogametocyte, **r** microgametocyte. **a**–**e**, **h**–**m**, **o**, **q** and **r** are *P. ovale curtisi* while **f**, **g**, **n** and **p** are *P. ovale wallikeri*

The youngest ring stages have a prominent round nucleus (Fig. 1a–c) that is often detached from the wisp of the cytoplasm, with a central vacuole (Fig. 1b, c). As this early stage progresses, the infected RBC becomes enlarged. Sometimes, the outline of the infected RBC changes to a slightly oval shape. Fimbriation may occur and the stippling starts to appear (Fig. 1a–c). The amount of cytoplasm increases as the parasite develops.

#### Trophozoite (Fig. 1d–g)

In the young trophozoite, the vacuole reduces in size and tends to disappear later. The cytoplasm becomes more abundant (Fig. 1d–f) and could appear as ‘pulled out’ or ragged (Fig. 1g). Sometimes it is possible to observe unusual elongated trophozoites that resemble the band form of *Plasmodium malariae*. The stippling (known as Schüffner stippling) increases and covers the whole infected RBC (Fig. 1f, g), which may begin to appear really oval in shape (Fig. 1d, f) with fimbriated edges (Fig. 1f). At the same time the pigment appears as a fine dust, like brown grains (Fig. 1f). As the parasite grows, these grains aggregate in dark brown beads (Fig. 1g) and occasionally appear greenish as light is reflected off them. In the late trophozoite, the nucleus and the cytoplasm increases significantly in size (Fig. 1g) and the parasite may occupy up to half of the RBC. The typical schüffnerization progresses to larger and larger but regular red/violet dots that sometime obscure the infected RBC (Fig. 1f, g).

#### Schizont (Fig. 1h–m)

The prominent nucleus that appears as a deep red-staining mass in a lighter matrix starts to divide into nuclei that remain noticeably large (Fig. 1h). At this time the markedly blue-staining cytoplasm of the trophozoite can fade (Fig. 1f) before turning more purplish-blue in the late stages (Fig. 1i–l). The chromatin in the growing schizont is at first condensed (Fig. 1i) before it divides (Fig. 1j, k) to produce about eight nuclei (Fig. 1l). At the same time, the pigment grains continue to come together, although with no special localization within the infected RBC (Fig. 1i–k), and tend to form a single yellowish-brown patch (Fig. 1m). The Schüffner dots are more abundant and may darken the infected RBC (Fig. 1k, l). At maturity (Fig. 1m), typically eight merozoites are formed (range from 4 to 16). Higher numbers of merozoites occur more often in patients that relapse and have chronic infection; they are less neatly arranged (Fig. 1l) compared to those observed in a *P. malariae* schizont.

#### Gametocytes (Fig. 1n–r)

These usually appear late during the infection. The young gametocyte is difficult to distinguish from the compact late trophozoite, but generally the pigment is coarser and darker (Fig. 1n). As growth proceeds the gametocyte increases in size to fill the infected RBC completely. The changes to the infected RBC induced by the gametocytes are similar to those described for the asexual stages. The macrogametocyte has a medium blue-stained cytoplasm, the nucleus is large, compact and eccentrically located, with a non-homogenous distribution of dark red and light red staining, and generally adopts a semi-lunar to oval shape (Fig. 1o–q). The stippling of this stage is usually prominent (Fig. 1o–q), stains red, and is more or less organized in a ring around the parasite. The cytoplasm of the microgametocyte is pinker (Fig. 1r), with a very large nucleus that can occupy almost half of the parasite consists of areas that are stained a darker red, surrounded by a diffuse area of chromatin that fades to pale pink (Fig. 1r). Obvious and deep red-staining Schüffner dots encircle the parasite. The dark brown or black granules of pigment in the gametocyte are scattered throughout the cytoplasm (Fig. 1p, q), but occasionally in macrogametocytes they have a tendency to come together in a rod shape (Fig. 1p).

### Differential diagnostic

**Fig. 2:**
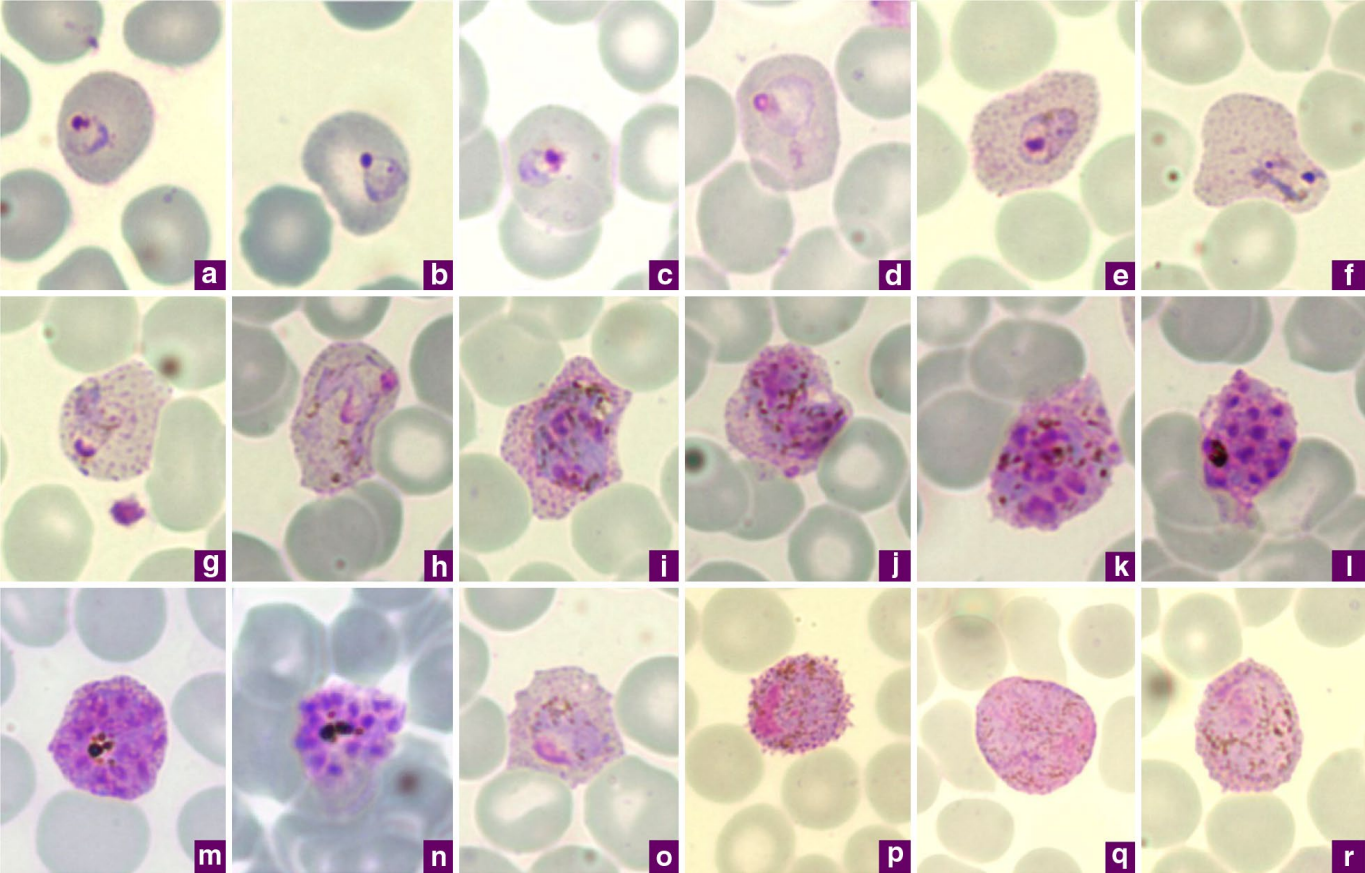
Microphotographs of *Plasmodium vivax* in Giemsa-stained thin blood films collected in Singapore. **a**, **b** ring stages, **c**–**e** young trophozoites, **f**–**h** amoeboid trophozoites, **i** young schizont,** j–l** growing schizonts, **m** developed schizont, **n** mature schizont, **o** young gametocyte, **p** macrogametocyte, **r**, **q** microgametocytes

In Giemsa-stained thin blood smears of the malaria parasite species usually found in humans (*P. falciparum*, *P. vivax*, *P. malariae*, *P. ovale*, and *P. knowlesi*), *P. ovale* could only be morphologically confused with *P. vivax*, the other tertian human malaria parasite that produces hypnozoite stages capable of inducing a relapse. In order to emphasize the differential diagnostic for *P. ovale* and *P. vivax*, comparative microphotographs of *P. vivax* are furnished in Fig. 2a–r. Additional details can be found in the comparison between the Donaldson strain of *P. ovale* with two strains of *P. vivax* [46].

In *P. ovale* infections, the infected RBCs are usually normal or just slightly enlarged (up to 1.25×), while those infected with *P. vivax* (Fig. 2a–r) are usually more enlarged (1.5× to up to 2×). The change in the shape of the infected RBC is another characteristic of *P. ovale*: a higher proportion of *P. ovale*-infected RBCs adopt a clear oval shape, whereas those infected by *P. vivax* display a distorted shape (Fig. 2e–j, m, o). The presence of infected RBCs with fimbriated edges, which seems to be linked to the smearing conditions, is indicative of *P. ovale* (Fig. 1a–c, f) where it occurs more frequently, but it can be observed for *P. vivax*. A detailed analysis of the changes in the outline of the infected RBCs can be found elsewhere [47].

In both *P. ovale* and *P. vivax*, the Schüffner dots are a good differential characteristic because in *P. ovale* the stippling appears and then covers the entire parasitized cell in the early stages (from young rings, Fig. 1a–d), while in *P. vivax* is appears later as the trophozoites develop (Fig. 2e–h). In addition, the stippling is often more pronounced than that observed in *P. vivax* and the dots are usually bigger, regular in size, red to violet in colour, and tend to darken the RBC.

Another element to consider is the parasite’s nucleus. In the *P. ovale* trophozoite (Fig. 1g), the nucleus is almost double the size of that observed in *P. vivax* at the equivalent stage (Fig. 2e–h), even though the *P. ovale* parasite is smaller in size. Furthermore, following the first steps of schizogony, the patches of chromatin are bigger in *P. ovale* than those produced in a young *P. vivax* schizont. In the mature schizont, the number of merozoites produced by *P. ovale* is usually eight (range from 4–16) (Fig. 1m) while *P. vivax* mature schizonts usually contain 16 merozoites (range 16–24) (Fig. 2n).

The gametocytes of *P. ovale* (Fig. 1o–r) are smaller in size than those of *P. vivax*, their cytoplasm has a lighter purple-blue to pink stain, and their pigment is less abundant than that of *P. vivax* (Fig. 2o–r).

While there are clear features that allow the distinction of *P. ovale* from *P. vivax* as presented above, we have not observed any morphological differences between *P. ovale curtisi* and *P. ovale wallikeri* by morphology.

### Molecular characterization of *Plasmodium ovale* from the cases imported in Singapore

In order to characterize the *P. ovale* parasites from the 11 cases imported in Singapore, genetic polymorphisms were sought on ten different loci spread across the three genomes of the parasites.

### Nuclear genes

#### *ssrRNA*

**Fig. 3:**
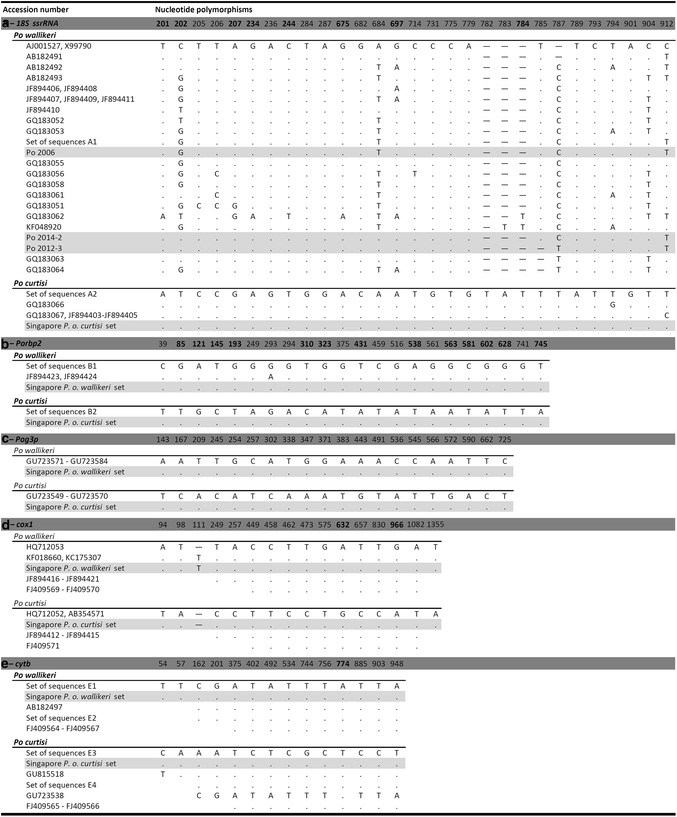
Dimorphic nature of *Plasmodium ovale curtisi* and *Plasmodium ovale wallikeri* based on the partial sequences from three nuclear and two mitochondrial genes. **a** 18S *ssrRNA*, **b** *Porbp2*, **c** *Pog3p*, **d** *cox1*, **e** *cytb* genes. Each alignment includes sequences from the *P. ovale* cases imported in Singapore (highlighted in *grey*) and from published sequences. Numbering is based for the 18S *ssrRNA* gene on the asexually expressed (A type) gene [L48987], for the *Porbp2* and *Pog3p* genes on the putative start-codons (ATG) identified by [23], for the *cox1* gene on the homologous position of the *P. falciparum* *cox1* gene [M76611], and for the *cytb* gene on the start-codon (ATG). For 18S *ssrRNA* gene the sets of sequences A1 and A2 contain: [L48986, L48987, AB182489, GQ231515, GQ183065, GQ183068] and [GU813972, JF894422, JF894425, JF894426] respectively; for *Porbp2* gene the sets of sequences B1 and B2 contain: [GU813972, JF894422, JF894425, JF894426] and [GU813971, JF894427-JF894429] respectively; for *cytb* gene the sets of sequences E1 to E4 gene contain [HQ712053, GQ231518-GQ231520], [GU723535-GU723537, GU723539-GU723548], [HQ712052, AB354571, GQ231516-GQ231517] and [AB182496, AF069625, GU723514-GU723534], respectively. The Singapore *P. o. wallikeri* and Singapore *P. o. curtisi* sets represent for each gene the sequences obtained from [Po 2006, Po 2012-3 and Po-2014-2] and from [Po 2003, Po 2012-1, Po 2012-2, Po 2012-4, Po 2013-1, Po 2013-2, Po 2013-3 and Po-2014-1], respectively. Non-synonymous mutations are in *boldface* type, *hyphens* represent gaps, *dots* represent nucleic acid identity and *blanks* represent unavailable information

This gene has long been used as a target for *Plasmodium* species identification, and the polymorphisms observed in this gene provided the first indication that *P. ovale* populations could be divided into ‘classical’ and ‘variant’ types. The sequences of the fragments (ca. 826 bp) amplified from the *P. ovale* samples from Singapore were compared (Fig. 3a; GenBank accession numbers KP050361 to KP050371) to those previously published [22, 23, 25, 48]. The parasites from eight samples were *P. o. curtisi* (Po 2003, Po 2012-1, Po 2012-2, Po 2012-4, Po 2013-1, Po 2013-2, Po 2013-3, and Po 2014-1), and the other three were *P. o. wallikeri* (Po 2006, Po 2012-3 and Po 2014-2). There was a higher diversity in the point mutations found for the *P. o. wallikeri* sequences, but the number of samples was too low to allow any meaningful conclusion.

#### *Porbp2*

The fragment (ca. 793 bp) analysed has been used previously to discriminate *P. o. wallikeri* from *P. o. curtisi* [23–25]. The sequences obtained for the 11 imported cases (Fig. 3b; GenBank accession numbers KP050394 to KP050404) fully concord with those published to date, and confirmed the species identification obtained using the ssrRNA locus.

#### *Pog3p*

The fragment (ca. 662 bp) analysed has been used previously to discriminate *P. o. wallikeri* from *P. o. curtisi* [23]. The sequences obtained for the 11 imported cases (Fig. 3c; GenBank accession numbers KP050383 to KP050393) fully concord with those published to data, and confirmed the species identification obtained using the ssrRNA locus.

#### *Potra*

**Fig. 4:**
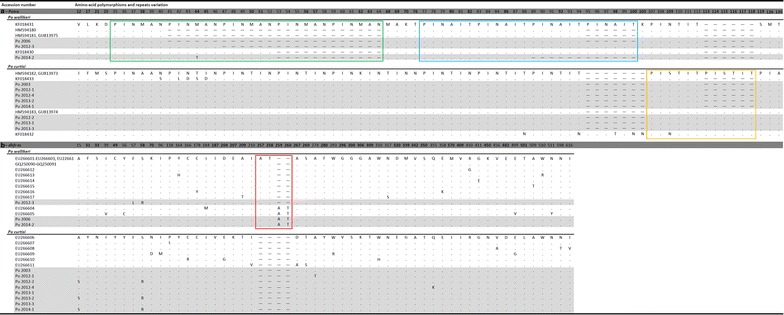
Dimorphic nature of *Plasmodium ovale curitsi* and *Plasmodium ovale wallikeri* based on the translated amino acid sequences from two additional nuclear genes. **a** *Potra* gene, and **b** *dhfr*-*ts* gene. Each alignment includes sequences from the imported *P. ovale* cases imported in Singapore (highlighted in *grey*) and from published sequences. Dimorphic sites are in *boldface* type. For *Potra* gene, the distinction of between *P. o. wallikeri* types 1 and 2 is based on a two amino acid variation located outside of the amplified fragment presented here [24], and the distinction between *P. o. curtisi* types 1 and 2 is based on the number of PISTIT repeat (n = 1 or 2) [23, 24]. For *dhfr*-*ts* gene, in addition to the dimorphisms between *P. o. wallikeri* and *P. o. curtisi*, the amino acid polymorphisms within each species are included to illustrate internal variations. In *Potra* gene for *P. o. wallikeri* the green and blue boxes represent the PINMAN repeats (n = 1–5) region and the PINAIT repeats (n = 2–4) region, respectively; for *P. o. curtisi* the *orange box* represents the PISTIT repeat (n = 1 or 2) region. In *dhfr*-*ts* the red box represents the AT repeats (n = 1 or 2) region. Numbering is based on the ORF identified by [24] for *Potra*, and on the putative start-codon (ATG) of *dhfr*-*ts*. *Hyphens* represent gaps and *dots* represent amino acid identity

The fragment of variable size (from 293 to 347 bp) that was analysed has been used previously to discriminate *P. o. wallikeri* from *P. o. curtisi* [23, 24, 49]. Comparison of the predicted amino acid sequences obtained for the 11 imported cases (Fig. 4a; GenBank accession numbers KP050372 to KP050382) confirmed the species identification obtained using the other loci. Furthermore, it showed that Po 2003, Po 2012-1, Po 2012-4, Po 2013-2, and Po 2014-1 are similar to *P. o. curtisi* type 1 [HM594182], while Po 2012-2, Po 2013-1 and Po 2013-3 are similar to *P. o. curtisi* type 2 [HM594183] which is distinguished by two 6-amino acid (PISTIT) repeat units [23, 24]. Two of the three *P. o. wallikeri* sequences showed novel genotypes differing from previously published genotypes by the size of the repeat units (*Pow type 1 [HM594180] and type 2 [HM594181]*).

#### *dhfr*-*ts*

Non-synonymous mutations and differences in the number of amino acid repeat units have been reported for the *dhfr*-*ts* of *P. o. curtisi* and *P. o. wallikeri* [23, 50]. Comparison of the complete gene sequences for the imported cases (GenBank accession numbers KP050405 to KP050415) with those published (Fig. 4b), confirmed the classification into two groups of eight *P. o. curtisi* and three *P. o. wallikeri* sequences, although some additional non-synonymous mutations were observed.

#### *asl*

**Fig. 5:**
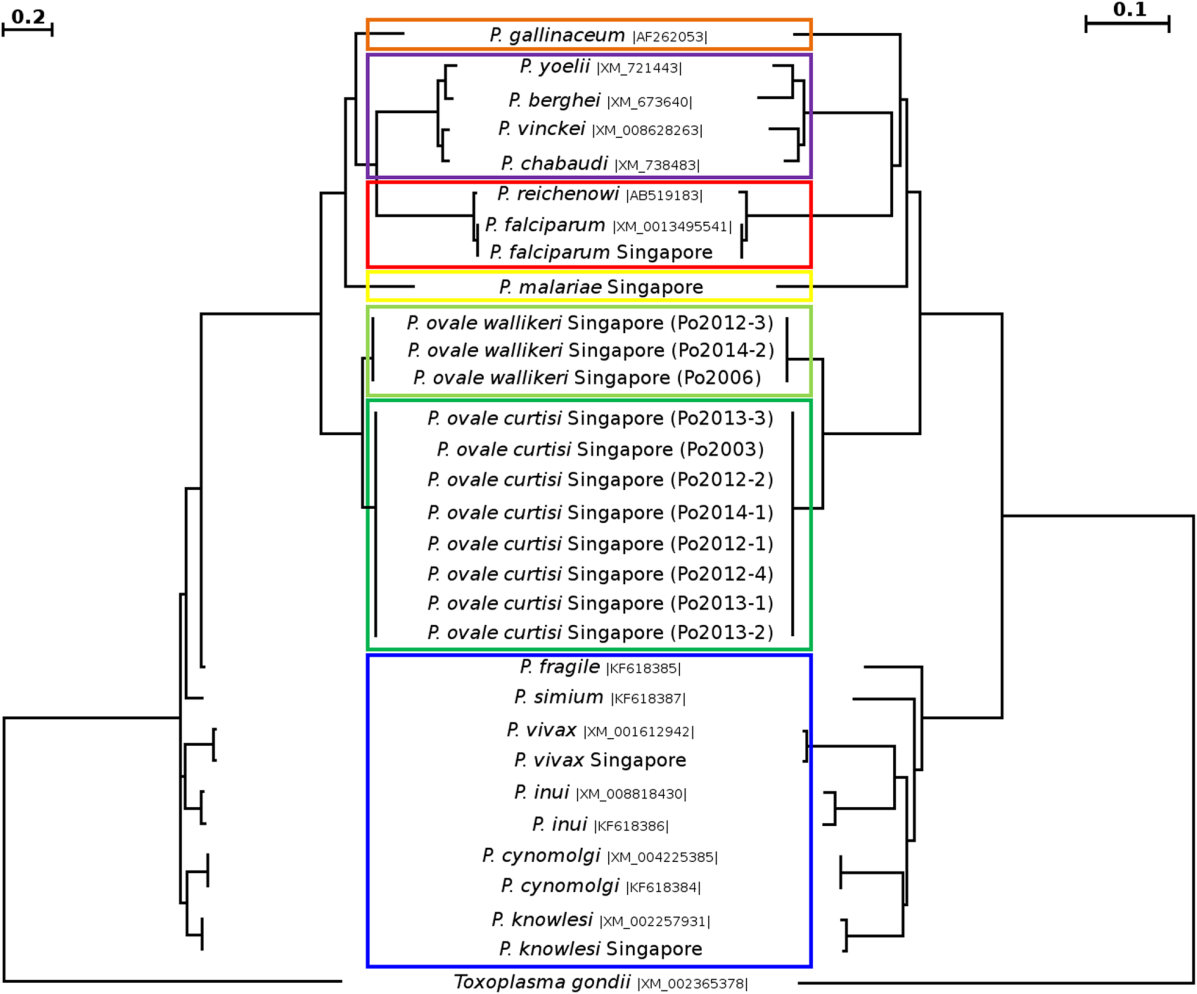
Combined phylogenetic trees generated by maximum likelihood (ML) (*left part*) and neighbour joining (NJ) (*right part*) methods using the partial sequences of the *asl* gene from different *Plasmodium* species. Of the sequences in the dataset, the 15 obtained in the present study are in boldface type (cases imported in Singapore: 8 *P. o. curtisi* (*dark green box*), 3 *P. o. wallikeri* (light green box), with 1 *P. vivax*, 1 *P. falciparum*, 1 *P. knowlesi*, and 1 *P. malariae*), the other 15 *Plasmodium* sequences (GenBank accession number is provided between vertical bars) from *Plasmodium* species that infect humans and primates (*P. falciparum* and *Plasmodium reichenowi*, *red box*; *P. malariae*, *yellow box*; *Plasmodium fragile*, *Plasmodium simiovale*, *Plasmodium inui*, *Plasmodium cynomolgi*, *P. knowlesi* and *P. vivax*, *blue box*), apes, rodents (*Plasmodium yoelii*, *Plasmodium berghei*, *Plasmodium vinckei*, and *Plasmodium chabaudi*, *purple box*) and birds (*Plasmodium gallinaceum*, *orange box*). The homologous fragment from the *Toxoplasma gondii* *asl* gene was used at the out-group to root the trees. After alignment the sequences have been trimmed (≈800 bp) to adjust their size to the fragment with the most coverage

**Fig. 6:**
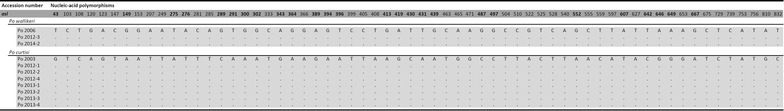
Dimorphic nature of *Plasmodium ovale curtisi* and *Plasmodium ovale wallikeri* based on the partial sequences from the nuclear gene *asl.* The alignment highlights differences between *P. o. curtisi* on *P. o. wallikeri* at 62 loci. Numbering is based on the first nucleic acid of the forward oligonucleotide primer. Non-synonymous mutations are in *boldface* type, and *dots* represent nucleic acid identity

This gene encodes the housekeeping protein adenylosuccinate lyase, an enzyme that is responsible for the salvage of host purines that are the used for DNA synthesis by *Plasmodium* [51]. The value of *asl* for phylogenetic and evolutionary studies of *Plasmodium* parasites has been noted previously [52–54]. Amplification of *asl* was carried out for the 11 *P. ovale* samples as well as for *P. vivax*, *P. falciparum*, *P. malariae*, and *P. knowlesi* samples from cases recorded in Singapore. A 841-bp fragment was obtained for all the samples, and all the sequenced derived (GenBank accession numbers KP050460 to KP050474) were added to published ones for the phylogenetic analyses (Fig. 5). Sequences for this gene were not hitherto available in GenBank for *P. ovale* and *P. malariae*. A total of 62 dimorphic loci have been identified between *P. o. curtisi* and *P. o. wallikeri* (Fig. 6). The number of dimorphic loci was higher than those recorded for the other genes studied in the present work. The two phylogenies obtained (Fig. 5) provided similar tree architecture, with all the *P. ovale* grouped within a biphyletic clade where *P. o. curtisi* and *P. o. wallikeri* are clearly separated.

### Mitochondrial genes

#### *cox1*

This gene is commonly used for phylogenetic and evolutionary studies, DNA bar-coding of metazoans, and to investigate *P. o. curtisi* and *P. o. wallikeri* [25, 26]. A 1,412-bp fragment was amplified from each sample (GenBank accession numbers KP050416 to KP050426) and their sequences were compared to published ones (Fig. 3d). The sequences from the 11 imported *P. ovale* cases matched perfectly with those published for *P. o. curtisi* and *P. o. wallikeri* for all the 15 dimorphic loci.

#### *cytb*

This gene is also commonly used for phylogenetic and evolutionary studies, and it has been used to discriminate between the two *P. ovale* species [23, 26]. A 1244-bp fragment was amplified from each of the imported *P. ovale* cases and their sequences (GenBank accession numbers KP050427 to KP050437) compared to those previously published (Fig. 3e), yielding the same conclusion as that from the *cox1* gene above.

### Apicoplast genes

Two conserved genes used for phylogenetic and evolutionary studies belong to the third genome of the parasite and are as yet uncharacterized for both the *P. ovale* species.

#### *tufA*

**Fig. 7:**
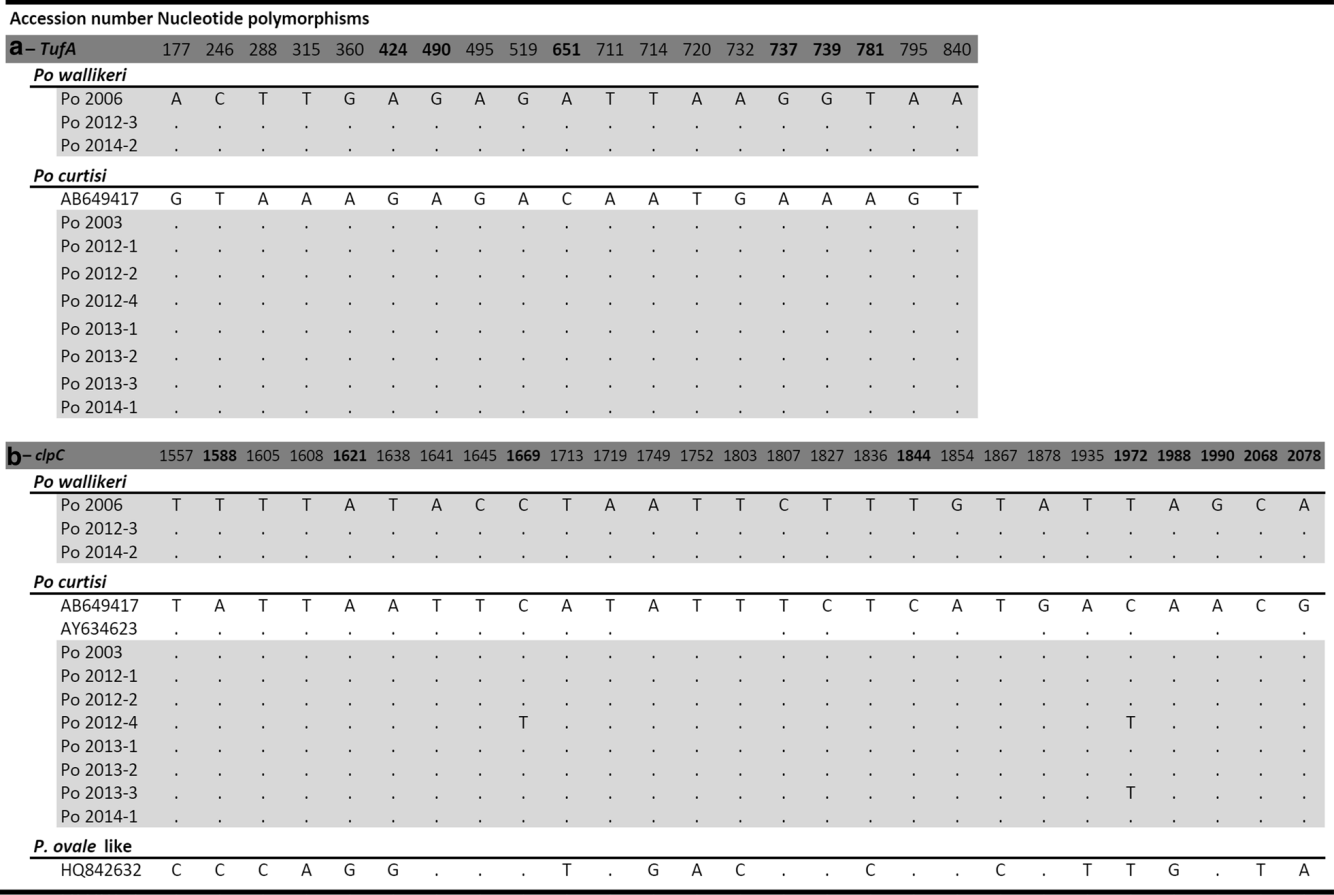
Dimorphic nature of *Plasmodium ovale curtisi* and *Plasmodium ovale wallikeri* based on the partial sequences from two apicoplast genes. **a** *tufA* gene, and **b** *clpC* gene. Each alignment includes the sequences from the *P. ovale* cases imported in Singapore (highlighted in *grey*) and published sequences. The *Plasmodium* *ovale*-like sequence [HQ842632] isolated from chimpanzee (Kaiser et al. unpublished) is included into this comparison but separated from *P. o. curtisi* and *P. o wallikeri* because it present 11 additional unique polymorphic loci beside the 15 dimorphic loci that distinguish *P. o. curtisi* and *P. o wallikeri*. Numbering is based on the start-codon (ATG) identified for both *tufA* and *clpC*. Non-synonymous mutations are in *boldface* type and *dots* represent nucleic acid similarity

A single full *P. ovaletufA* sequence has been deposited in GenBank [AB649417] [55]. It originates from the Nigeria II strain that is classed as a *P. o. curtisi*, and has been compared to the sequences (GenBank accession numbers KP050449 to KP050459) of the 814-bp fragment amplified from each of the imported cases (Fig. 7a). A total of 19 dimorphic loci were found between *P. o. curtisi* and *P. o. wallikeri*.

#### *clpC*

Three *clpC* sequences are available for *P. ovale* in GenBank: the full sequence extracted from [AB649417] [55], a partial sequence [AY634623] [56], and a sequence [HQ842632] (Kaiser et al. unpublished) from a *P. ovale*-like parasite detected in chimpanzee. These were compared to the sequences (GenBank accession numbers KP050438 to KP050448) of the 625-bp fragments amplified from the *P. ovale* imported cases (Fig. 7b). A total of 15 dimorphic loci were found between *P. o. curtisi* and *P. o. wallikeri*. Sequences [AB649417] and [AY634623] matched the *P. o. curtisi* parasites. However, for sequence [HQ842632], the variations were more diverse: of the 15 dimorphic loci, nine were similar to those of *P. o. curtisi*, four to those of *P. o. wallikeri*, and the remaining two loci differed from those previously noted for *P. o. curtisi* and *P. o. wallikeri*. Eleven additional mutations, three of which were non-synonymous, not previously observed in either *P. o. curtisi* and *P. o. wallikeri*, were also noted.

## Discussion

The microscopic morphology of the parasite in stained blood smears is still considered to be the gold standard for the identification of *Plasmodium* species, and it is often the only technique available in clinical laboratories. However, the subtle morphological characteristics that help distinguish *P. vivax* and *P. ovale* are readily missed by less experienced microscopists, and might even elude expert microscopists who only encounter one or other of these two species infrequently or when the quality of the smears or the staining are sub-optimal [15, 38, 57]. Thus, it was not surprising that all the confirmed *P. ovale* cases in Singapore had been misidentified at *P. vivax* by the hospital laboratory staff that encounter *Plasmodium* parasites relatively infrequently. Current rapid diagnostic tests (RDTs) are inadequate for the direct identification of *P. ovale* [58] although some might be helpful in preventing confusion with *P. vivax* [59]. Accurate diagnosis of *P. ovale* remains dependent on the use of molecular techniques such as PCR. However, even the reliable and well-adopted protocols [39] used in the present study has been shown to miss certain *P. ovale* cases [60]. To prevent the risk of overlooking *P. ovale*, especially in mixed infection with others malaria species one might consider to choose a more sensitive protocol [60, 61]. It should be noted that the misidentification of *P. ovale* as *P. vivax*, or vice versa, has little clinical consequence because the recommended treatment for both species is the same [62].

The imported *P. ovale* cases uncovered in this study were genetically characterized for markers (*ssrRNA*, *Potra*, *Porbp2*, *Pog3p*, *dhfr*-*ts, cytb*, *cox1*) used in other recent studies [23–26]. The data were concordant with those previously obtained and allowed them to be clearly identified as *P. o. curtisi* or as *P. o. wallikeri*. For the *ssrRNA*, *dhfr*-*ts* and *Potra* genes, synonymous and non-synonymous polymorphisms were noted within the sequences obtained for the *P. o. curtisi* or the *P. o. wallikeri* samples. These variations were broadly more numerous for the *P. o. wallikeri* sequences; however, a higher number of samples will be required to confirm this trend. These observations indicate that ‘sub-types’ of *P. o. curtisi* and *P. o. wallikeri* might occur [23, 24, 26, 50, 63, 64] in humans and in chimpanzees. Analysis of a larger number of samples from humans and chimpanzees will be needed to test the hypothesis that a ‘*P. ovale* species complex’ is shared between humans and apes.

In the present study the genetic characterization was extended to three other genes that proved to be dimorphic between the two *P. ovale* species. The nuclear *asl* gene (encoding adenylosuccinate lyase) had the highest number of dimorphic loci in comparison with the other genes analysed, and displayed no internal variations within either the *Poc* or the *Pow* samples. These observations are consistent with the notion that *P. o. curtisi* and *P. o. wallikeri* have been evolving separately over a long period of time [23]. The other two genes analysed (*tufA* and *clpC*) are present on the apicoplast, whose genome has only been previously characterized for the Nigeria II *P. ovale* isolate [56]. A phylogenetic analysis of the *asl* fragments built from the 15 sequences obtained in this study from cases imported into Singapore and from other sequences from various *P. ovale* and other *Plasmodium* species (downloaded from GenBank) that infect humans, apes, non-human primates, rodents, and birds showed that both *P. ovale* species cluster into a biphyletic clade that is related to other *Plasmodium* species of primates. This observation, based on a single nuclear gene, was congruent with the conclusions derived from analyses of the mitochondrial genome [26, 65, 66], but they did not support the recent suggestion, based on the analyses of the apicoplast genome, that *P. ovale* is closely related to the *Plasmodium* species of African rodents [56]. It also emphasizes that it would be desirable to adopt an approach based on multiple genes across several genomes, using a larger number of samples for each species, in order to increase the likelihood of deriving a true phylogenetic relationship between the various *Plasmodium* species [45].

All the confirmed *P. ovale* cases discovered in Singapore were imported from endemic areas for this species [12] from eight countries (six in Africa and two in South and Southeast Asia) where this parasite has been previously recorded: Nigeria [67], Central African Republic [68], Uganda [10, 69], Ivory Coast [70], Cameroon [71], Equatorial Guinea [72], Liberia [6], India [72, 73], and Indonesia [74, 75]. The one case imported from India is interesting in that although *P. ovale* was first reported in this country 75 years ago, albeit as a doubtful mixed infection with *P. falciparum* [72], it has been reported since only five times [73, 76–79]. It is not at present clear whether this suggests that this parasite species is on the increase in India or whether its prevalence has been grossly underestimated through misidentification as *P. vivax*. The case imported from Indonesia is equally interesting in that the patient had probably acquired the infection in Sumatra where *P. ovale* had not previously been recorded [5, 75].

*Plasmodium vivax* is consistently the species most frequently imported in Singapore (70 %, 1512 of the 2155 cases recorded between 2001 and 2014). Until the present study *P. ovale* infections had not been officially reported in Singapore, although a suspected diagnosis was made on two occasions in 1998 and in 2013 but not confirmed by MRC. For the period 2009–2011, molecular analysis of the 511 malaria-positive samples (386 identified as *P. vivax* by morphology) did not reveal any *P. ovale* infections, whereas nine *P. ovale* infections (four in 2012, three in 2013 and two in 2014) were detected from all the cases recorded in the last three years (316 cases with 238 identified as *P. vivax* by morphology in 2012–2014). For the period 2001–2008, a total of 1328 malaria-positive cases (888 identified as *P. vivax* by morphology) were notified to MOH, but only 226 frozen blood samples were available from this period (150 were identified as *P. vivax* by morphology) for molecular testing, revealing that two were actually *P. ovale* (one in 2003 and another in 2006). If one assumes the same proportion of misidentified *P. ovale* for the remaining 1,102 samples, then the expected total number of potential *P. ovale* cases for the 2001–2011 period would be 12 out of the 1,835 recorded cases. Thus, it is possible that there has been a sharp increase in the proportion of *P. ovale* recently imported to Singapore (an estimated 0.65 % for 2001–2011 versus 2.85 % for 2012–2014). Such an increase would be puzzling given the constant decrease in the total number of malaria cases recorded in Singapore since 2010 [80]. This pattern does not seem to be restricted to Singapore, with similar, recent reports of *P. ovale* in imported malaria cases among soldiers deployed in endemic areas [16, 81], tourists visiting endemic countries [82, 83], and foreign workers returning from endemic regions [84–86]. Several hypotheses, alone or in combination, might be for formulated to explain such an increase: (1) an upsurge of studies employing molecular tools that allow sensitive and accurate detection and identification of *Plasmodium* species (this would not apply to the cases imported to Singapore over the last 6 years, because all were molecularly tested); (2) an increase over recent years in the number of travellers, visitors or migrant workers to *P. ovale*-endemic countries; and, (3) a recent increase in the prevalence of *P. ovale* in the endemic areas, possibly as a result of extensive campaigns directed against *P. falciparum* that might favour this less common species as suggested by [15] or as observed with the recent increase of *P. knowlesi* in Malaysia [87].

### Public health concern in Singapore

*Plasmodium ovale* can induce relapses that can occur a long time after the primary infection and over several years [5, 9, 88]. Six of the nine imported cases to Singapore in recent years were clearly due to relapsing *P. ovale* that had been acquired a few months to 4 years previously. Given the presence of competent mosquito vectors such as *Anopheles maculatus* in Singapore [89] and the possibility of relapse episodes long after the primary infection [5, 9, 88] some of which are potentially asymptomatic, clinicians, epidemiologists, laboratory technicians, and public health officials should place *P. ovale* on a par with *P. vivax* as a potential threat to malaria elimination programmes [31, 82, 90, 91].

## Conclusion

Routine malaria surveillance, incorporating molecular species determination performed by the MRC-NPHL, detected nine *P. ovale* infections that had been misidentified in clinical laboratories settings as *P. vivax* among malaria cases imported to Singapore over the last 3 years. This is the first confirmed report of *P. ovale* in Singapore. Retrospective study on archived blood samples identified two additional misidentified *P. ovale* infections: one in 2003 and another in 2006. Both *P. o. curtisi* and *P. o. wallikeri* were found among the cases imported to Singapore, and their genetic dimorphism was confirmed using ten markers spread across the parasites’ three genomes (seven previously analysed in other studies and three additional ones that were not characterized before). The data suggest that there might have been an apparent increase in the proportion of imported *P. ovale* in the face of a decrease in the overall number of malaria cases recorded in Singapore. Given its ability to cause relapses, *P. ovale* should not be neglected as a public health concern.

## Additional file

10.1186/s12936-015-0985-8 Details about misidentifications, travel histories, onsets and symptoms of the *Plasmodium ovale* cases recorded in Singapore.
