# Supplementary material for: Molecular characterization of misidentified Plasmodium ovale imported cases in Singapore
Source: Malar J. 2015 Nov 14;14:454. doi: 10.1186/s12936-015-0985-8 (PMC4650842; doi:10.1186/s12936-015-0985-8)
Supplement: Supplementary file 1 — 10.1186/s12936-015-0985-8 Details about misidentifications, travel histories, onsets and symptoms of the Plasmodium ovale cases recorded in Singapore. [file 12936_2015_985_MOESM1_ESM.doc]

**Supplement 1**

Details about the misidentifications, travel histories, onsets and symptoms of the *P. ovale* cases recorded in Singapore.

| Case | Laboratory  report | MRC  report | Case history |
| --- | --- | --- | --- |
| Case 1998 | *P. falciparum*  + *P. ovale*? | N.A.^Δ‡^ | Imported malaria involving a 30-year-old Singaporean patient. Parasite load was 0.45%. |
| Case  2003 | *P. vivax* | *P. ovale curtisi* | Imported malaria involving a 53-year-old Singaporean patient who travelled to India (Uttar Pradesh) from 22 Feb - 16 Mar 2003 for missionary/volunteer work. Parasite load was 0.25%. |
| Case  2006 | *P. vivax* | *P. ovale wallikeri* | Imported malaria involving a 19-year-old Nigerian patient who arrived from Nigeria to Singapore on 28 Oct 2006 for studying. Parasite load was 0.03%. |
| Case  2012-1 | *P. vivax* | *P. ovale curtisi* | Imported malaria involving a 41-year-old Singaporean patient who visited Nigeria from 4 May - 4 Dec 11. He developed fever, chills, rigors, headache and body ache on 6 Jan 12. Parasite load was 0.2%. |
| Case  2012-2 | *P. vivax*  (morphology)  *P. ovale**  (PCR) | *P. ovale curtisi*  *P. ovale curtisi* | Imported malaria involving a 22-year-old Indonesian patient who travelled to Sumatra, Indonesia from 3 - 8 Jan 12.  He developed fever, chills, rigors, body ache and headache on 28 Jan 12, saw a GP and was admitted to hospital on 9 Feb 12. Parasite load was 0.35%. Two samples were taken from this patient and processed by different labs. The case did not remember having had malaria before. |
| Case  2012-3 | *P. vivax* | *P. ovale wallikeri* | Imported malaria involving a 20-year-old Singaporean patient who visited Uganda from 3 Dec 11 - 15 Jan 12 for volunteer work. She developed fever, chills, rigors and headache on 20 Dec 11 and self-medicated. Her symptoms resurfaced on 22 and 28 Apr 12 and sought outpatient treatment at GP on two occasions and subsequently was admitted to hospital from 28-2 May 12. Parasite load was 0.7%. |
| Case 2012-4 | *P. vivax* | *P. ovale curtisi* | Imported relapsing malaria involving a 38-year-old Filipino patient who arrived from Central African Republic on 14 Jan 12. She developed symptoms on 25 Oct 12 and was hospitalized on 29 Oct 12. Prior to her onset of illness, she also visited the Philippines from 21 Jan – 14 Mar 12 and Malaysia from 4-8 Jun 12. The case had a history of malaria in Central African Republic in Nov 2011. |
| Case 2013-1 | *P. vivax* | *P. ovale curtisi* | Imported relapsing malaria case involving a 24-year-old French patient who arrived on 25 Oct 12 from Ivory Coast. Prior onset she visited Indonesia from 30 Nov-2 Dec 12. She developed fever, headache, vomiting, cough and abdominal pain on 28 Dec 12 and was hospitalized on 3 Jan 13. Parasite load was 0.1%. She had a history of malaria in Feb 12 in Ivory Coast. |
| Case 2013-2 | *P. vivax* | *P. ovale curtisi* | Imported relapsing malaria case involving a 40-year-old Singaporean who visited Cameroon from 1 Nov-2 Dec 12. He developed fever on 4 May 13 and sought outpatient treatment on 8 May 13. Parasite load was 0.2%. The case has a history of Malaria in Cameroon in Nov 12. |
| Case 2013-3 | *P. vivax* | *P. ovale curtisi* | Imported relapsing malaria case involving a 48-year-old Chinese patient who arrived on 18 Apr 13. He developed fever, body ache, nausea, myalgia and lethargy on 8 Aug 13 and was admitted to SGH on 17 Aug 13. Parasite load was 0.02%. Case had malaria in Liberia in 2009. |
| Case 2014-1 | *P. vivax* | *P. ovale curtisi* | Imported relapsing malaria case involving a 46-year-old Chinese patient who arrived on 29 Nov 13 from China. Case has a history of malaria in 2012 & 2013 when working in Equatorial Guinea. |
| Case 2014-2 | *P. vivax* | *P. ovale wallikeri* | Imported relapsing malaria case involving a 34-year-old Chinese patient who arrived from China on 27 Mar 14. He developed fever, chill & rigors and headache on 7 Jul 14. Parasite load was 0.1%. Case had a history of malaria in 2012 and 2013 when working in Liberia and sought outpatient treatment. |

^Δ^ cases not confirmed by MRC in 1998. ^‡^ No more material available to reinvestigate this case.

* Second sample collected for this case and correctly identified by PCR in a second laboratory.
